# Tunable Intranasal Polymersome Nanocarriers Triggered Olanzapine Brain Delivery and Improved In Vivo Antipsychotic Activity

**DOI:** 10.3390/pharmaceutics17070811

**Published:** 2025-06-23

**Authors:** Ahmed A. Katamesh, Hend Mohamed Abdel-Bar, Rania Mahafdeh, Mohammed Khaled Bin Break, Shimaa M. Hassoun, Gehad M. Subaiea, Mostafa E. El-Naggar, Khaled Almansour, Hadel A. Abo El-Enin, Heba A Yassin

**Affiliations:** 1 Department of Pharmaceutics, College of Pharmacy, University of Ha’il, Ha’il 81442, Saudi Arabia; 2 Department of Pharmaceutics, Faculty of Pharmacy, University of Sadat City, Menoufia 32897, Egypt; 3 Department of Clinical Pharmacy and Therapeutics, Faculty of Pharmacy, Jadara University, Irbid 21110, Jordan; r.mahafdeh@jadara.edu.jo; 4 Department of Pharmaceutical Chemistry, College of Pharmacy, University of Ha’il, Ha’il 81442, Saudi Arabia; 5 Medical and Diagnostic Research Centre, University of Ha’il, Ha’il 55473, Saudi Arabia; 6 Department of Pharmacology, College of Pharmacy, University of Ha’il, Ha’il 81442, Saudi Arabiag.subaiea@uoh.edu.sa (G.M.S.); 7 Department of Pharmacology and Toxicology, Faculty of Pharmacy, University of Sadat City, Menoufia 32897, Egypt; mostafa.elnaggar@fop.usc.edu.eg; 8 Department of Pharmaceutics, Egyptian Drug Authority, Giza 12511, Egypt; hadelaboenin@outlook.com; 9 Department of Pharmaceutics and Pharmaceutical Technology, Faculty of Pharmacy, Pharos University in Alexandria, Alexandria 21648, Egypt

**Keywords:** intranasal, polymersomes, olanzapine, antipsychotic, brain targeting

## Abstract

**Background**: Olanzapine (Ola) is a second-generation antipsychotic with clinical utility limited by poor brain bioavailability due to blood–brain barrier restriction, hepatic first-pass metabolism, and systemic side effects. This study aimed to develop and optimize a novel intranasal polymersome-based nanocarrier (Poly_Ola_) to enhance brain targeting, therapeutic efficacy, and safety of Ola. **Methods**: Poly_Ola_ was prepared using poloxamer 401 and optimized through a Box–Behnken Design to minimize particle size and maximize entrapment (EE%) and loading efficiency (LE%). The formulation was characterized by size, morphology, drug release, and serum stability. In vivo studies in adult male Sprague-Dawley rats assessed pharmacokinetics (plasma and brain concentrations), pharmacodynamic efficacy in a ketamine-induced schizophrenia model, and systemic safety markers including metabolic, hepatic, and testicular oxidative stress indicators. **Results**: Optimized Poly_Ola_ exhibited a particle size of 78.3 ± 4.5 nm, high EE% (91.36 ± 3.55%), and sustained in vitro drug release. It remained stable in serum for 24 h. Intranasal administration significantly improved brain delivery of Ola, achieving a 2.7-fold increase in C_max_ and a 5.7-fold increase in AUC compared to oral dosing. The brain T_max_ was 15 min, with high drug-targeting efficiency (DTE% = 365.38%), confirming efficient nose-to-brain transport. Poly_Ola_-treated rats showed superior antipsychotic performance, reduced extrapyramidal symptoms, and improved systemic safety evidenced by mitigated weight gain, glycemic control, normalized liver enzymes, and reduced oxidative stress. **Conclusions**: Poly_Ola_ offers a safe and effective intranasal delivery platform for Ola, enabling targeted brain delivery and improved management of schizophrenia with reduced peripheral toxicity.

## 1. Introduction

Schizophrenia is a severe and long-lasting neuropsychiatric disorder that impacts nearly 1% of people worldwide. The disorder manifests through a diverse spectrum of clinical features, typically categorized into positive symptoms (such as hallucinations and delusions), negative symptoms (including anhedonia and social withdrawal), and cognitive deficits (encompassing impairments in memory, attention, and executive functioning) [1,2]. Its complex etiology—rooted in genetic, environmental, and neurodevelopmental factors—renders the disease particularly challenging to manage, placing a substantial burden on patients, caregivers, and global healthcare systems [3].

Among second-generation antipsychotics (SGAs), olanzapine (Ola) has emerged as a leading therapeutic agent due to its robust efficacy in alleviating positive symptoms through the modulation of dopaminergic and serotonergic neurotransmission [4]. However, despite its widespread clinical use, Ola’s therapeutic application is fraught with considerable limitations. While moderately effective against positive symptoms, it offers limited benefit for the persistent negative and cognitive symptoms that critically impair functional outcomes [5]. Additionally, the pharmacokinetic and pharmacodynamic properties of Ola pose further challenges. Notably, its limited ability to cross the blood–brain barrier (BBB) primarily due to its role as a substrate for p-glycoprotein coupled with extensive first-pass hepatic metabolism results in suboptimal brain delivery and low bioavailability. More importantly, chronic Ola therapy is strongly associated with serious metabolic and systemic toxicities, including significant weight gain, insulin resistance, hyperglycemia, dyslipidemia, and hepatic and reproductive dysfunctions. These factors significantly reduce its therapeutic efficacy, hindering its full potential in the treatment of schizophrenia [6]. Attempts to address these issues have included oral sustained-release and long-acting injectable formulations, such as Ola pamoate, which are designed to improve patient adherence and maintain therapeutic plasma levels over extended periods [7,8]. However, these strategies largely fail to resolve the fundamental issues of inadequate BBB penetration and excessive peripheral exposure, which continue to underlie the drug’s unfavorable safety profile [9].

Nanotechnology-based drug delivery systems have gained considerable attention as transformative tools in central nervous system (CNS) pharmacotherapy. These nanosystems are engineered to enhance drug solubility, stability, and targeted biodistribution, offering a compelling solution to the inherent limitations of conventional antipsychotic formulations [9,10]. Several types of nanocarriers including lipid-based nanoparticles, polymeric micelles, and polymeric nanoparticles have demonstrated promise in enhancing the brain uptake and therapeutic effectiveness of olanzapine while reducing systemic toxicity [11,12,13,14,15,16]. Among these nanocarriers, polymersomes (Poly) are particularly promising due to their exceptional structural stability, biocompatibility, and versatile properties, which can be finely tuned for effective drug encapsulation and controlled release. These characteristics make Poly an ideal candidate for targeted drug delivery, especially for applications requiring precise control over the drug release profile [17]. Poly are vesicular nanostructures that arise from the spontaneous self-assembly of amphiphilic block copolymers in aqueous media. They offer several advantages over traditional liposomes, including enhanced mechanical stability, higher drug-loading capacities, and the ability to encapsulate both hydrophilic and hydrophobic compounds. These properties make Poly highly effective for a wide range of drug delivery applications, particularly in complex therapeutic scenarios [18,19]. Moreover, the versatile surface functionalization of Poly enables precise targeting of therapeutic agents to the brain. This capability allows for more effective treatment with reduced systemic side effects, improving the overall therapeutic potential of the drug [20]. Additionally, the physicochemical properties of Poly such as particle size, surface charge, and membrane thickness can be precisely tailored to optimize the delivery and release of Ola within the brain. By adjusting these characteristics, Poly can be engineered to improve brain penetration, enhance drug stability, and control the release profile, ultimately maximizing the therapeutic efficacy of Ola while minimizing side effects [21,22]. Encapsulating Ola within Poly nanocarriers offers several significant advantages. First, it provides increased protection of the drug from premature degradation and metabolism, thereby enhancing its bioavailability. Second, it enables a controlled and sustained-release profile, which can reduce the frequency of dosing and improve therapeutic outcomes. Additionally, the surface of these nanocarriers can be modified with brain-targeting ligands, facilitating enhanced BBB penetration and enabling precise drug delivery to specific sites within the CNS. These features contribute to a more effective and targeted therapeutic strategy for treating schizophrenia [23]. One promising approach to further enhance drug delivery to the brain is intranasal (IN) administration, which is gaining attention as a non-invasive route for CNS drug delivery. The nasal route provides a direct conduit to the brain through the olfactory and trigeminal nerve pathways, enabling circumvention of the blood–brain barrier and promoting rapid drug transport to the central nervous system [24,25]. This route not only facilitates efficient drug targeting to the CNS but also minimizes systemic exposure, thereby reducing the risk of peripheral side effects typically associated with the systemic administration of antipsychotics [26]. Studies have highlighted the potential of IN delivery to enhance brain uptake of various nanocarriers, including liposomes and polymeric nanoparticles. This underscores the route’s promise for improving drug delivery to the brain, particularly for the treatment of neuropsychiatric disorders like schizophrenia [27,28,29]. Therefore, when combined with the advantages of Poly nanocarriers, intranasal administration could further optimize Ola delivery, ensuring both targeted and efficient brain penetration.

Given these considerations, the present study aimed to develop and optimize an IN polymersome-based Ola formulation (Poly_Ola_) for enhanced brain delivery and improved therapeutic outcomes in schizophrenia. A Box–Behnken design (BBD) was applied to methodically refine formulation variables, targeting an optimal formulation profile characterized by minimal particle size, high entrapment efficiency, and maximal drug loading. The prepared Poly_Ola_ formulation was characterized for its physicochemical properties and in vitro release behavior. Its pharmacokinetic profile was evaluated in rats following IN administration, with a focus on brain bioavailability and targeting efficiency. In addition, the antipsychotic efficacy was assessed using a ketamine-induced schizophrenia-like model, and systemic safety was investigated through metabolic, hepatic, and reproductive markers, including oxidative stress indices in testicular tissue.

## 2. Materials and Methods

### 2.1. Materials

Olanzapine (Ola), poloxamer 401 (P401), phosphate-saline buffer (PBS), dimethyl sulfoxide (DMSO), dimethyl formamide (DMF), Acetonitrile (HPLC grade), phosphoric acid, triethylamine (HPLC grade) and fetal bovine serum (FBS) were supplied from Sigma Aldrich, Dorset, UK. Tween 80 was purchased from Fluka Chemika-BioChemika, Buchs, Switzerland.

### 2.2. Method

#### 2.2.1. Formulation of Olanzapine-Loaded Polymersomes

Polyformulations were prepared using different concentrations of Poloxamer 401 (P401) and olanzapine (Ola). Specifically, P401 was dissolved at concentrations of 30, 40, and 50 mg/mL, and Ola was incorporated at 1, 2, and 3 mg/mL, each in 10 mL of phosphate-buffered saline (PBS, pH 7.4). The mixtures were stirred using a magnetic stirrer (CB302, Jenway Ltd., Essex, UK) at 500, 750, or 1000 rpm for 1 h at 4 °C. Subsequently, the dispersions were maintained under continuous stirring at room temperature for an additional 4 h [30]. The resulting colloidal formulations were purified via ultrafiltration centrifugation at 17,762× *g* for 45 min at 4 °C using Amicon™ Ultra-15 centrifugal filter units (MWCO 30 kDa, Merck, Frankfurter, Germany). The supernatant containing free drug was carefully removed, and the retained vesicles were washed twice with PBS to eliminate residual unencapsulated Ola. The final purified Poly_Ola_ samples were reconstituted in PBS and stored at 4 °C until further analysis.

#### 2.2.2. Optimization of Olanzapine-Loaded Polymersomes

To statistically optimize the preparation variables of Poly_Ola_, a three-factor, three-level BBD was employed using the Design Expert software (Version 13, Stat-Ease Inc., Minneapolis, MN, USA). The effects of three variables P401 concentration (A), Ola concentration (B), and stirring speed (C) on the outcomes, particle size (Y1), entrapment efficiency (EE%) (Y2), and loading efficiency (LE%) (Y3), were examined. The study aimed to determine how these factors influence Poly_Ola_ characteristics, with a focus on minimizing particle size and maximizing both EE% and LE%. The optimal Poly_Ola_ formulation was chosen based on calculated desirability and then prepared in triplicate to validate the statistical model before conducting further investigations. Details of the independent variable levels and dependent responses are provided in Table 1.

#### 2.2.3. In Vitro Characterization of Olanzapine-Loaded Polymersomes

##### Particle Size and Zeta Potential Measurement

Particle size and polydispersity index (PDI) of different Poly_Ola_ formulations were measured using dynamic light scattering (DLS) on a Nanosizer ZS Series instrument (Malvern Instruments, Southborough, MA, USA). Zeta potential was evaluated via electrophoretic light scattering. Briefly, each formulation was diluted 1:100 (*v*/*v*) with deionized water and introduced into disposable folded capillary Zeta cells. All measurements were performed at 25 °C, with each value representing the mean of 20 runs, and each run was conducted in triplicate to ensure reproducibility [13].

##### Determination of Olanzapine Entrapment Efficiency and Loading Efficiency

The entrapped Ola content in the Poly_Ola_ formulations was quantified using a validated high-performance liquid chromatography (HPLC) method [31]. Briefly, 0.5 mL of the formulation was dissolved in a 50 mL mixture of dimethyl sulfoxide (DMSO) and dimethylformamide (DMF) in a 1:1 (*v*/*v*) ratio, followed by magnetic stirring at 500 rpm for 30 min. The resulting solution was centrifuged at 1108× *g* for 15 min, and the Ola concentration in the supernatant was analyzed. HPLC analysis was conducted using an Agilent 1100 system equipped with a quaternary pump, autosampler, and variable-wavelength detector, Waldbronn, Germany. Chromatographic separation was achieved on a LiChrospher^®^ 60 RP column (250 × 4.6 mm, 5 μm) maintained at 25 °C, with detection at 270 nm. The mobile phase consisted of deionized water containing 0.25% phosphoric acid and 0.05% triethylamine, mixed with acetonitrile in an 86:14 (*v*/*v*) ratio, and delivered at a flow rate of 1 mL/min. To determine LE%, an aliquot of purified Poly_Ola_ dispersion (1 mL) was freeze-dried using a laboratory lyophilizer (BENCHTOP Manifold freeze dryer, Millrock Technology, Inc., Kingston, NY, USA). Samples were pre-frozen at −40 °C for 3 h, followed by primary drying under vacuum (100 mTorr) at −40 °C for 24 h, and secondary drying at 25 °C for 8 h. The resulting dry residue was weighed to determine the total weight of the Poly_Ola_.

The EE% and LE% were calculated according to the following equations:

(1) $$EE\%=\frac{Ola\;amount\;quantified\;in\;the\;Poly}{Initial\;amount\;of\;Ola\;added}\times 100$$

(2) $$LE\%=\frac{Weight\;of\;Ola\;entrapped\;in\;the\;Poly}{Weight\;of\;Ola\;loaded\;Poly}\times 100$$

##### Transmission Electron Microscopy

The optimized Poly_Ola_ nanovesicle formulation’s morphology was characterized using a transmission electron microscope (TEM, Joel JEM 1230, Tokyo, Japan). A drop of the sample was applied onto a carbon-coated copper grid to form a thin film, followed by negative staining with 1% phosphotungstic acid solution [13].

##### In Vitro Serum Stability

The impact of serum on the physicochemical properties of Poly_Ola_ was evaluated by assessing particle size, polydispersity index (PDI), and zeta potential following incubation with 10% and 50% (*v*/*v*) fetal bovine serum (FBS) for 4, 24, and 48 h at 37 ± 0.5 °C [32].

##### In Vitro Drug Release

The in vitro release rate of Ola from the optimized Poly_Ola_ was quantified using the dialysis method [13]. A sample containing Poly_Ola_ equivalent to 2 mg of Ola was diluted with 1 mL of simulated nasal fluid (SNF, pH 7.4) and transferred into a dialysis membrane with a molecular weight cut-off of 10 kDa. The tightly sealed dialysis bag was suspended in 25 mL of SNF (pH 7.4) and maintained at 35 ± 0.5 °C, the reported nasal mucosa temperature in a thermostatically controlled shaking water bath (Daihan Labtech Shaker Water Bath, LSB 030S, Seoul, Republic of Korea) operating at 100 ± 0.1 strokes/min [33]. At predetermined time intervals up to 24 h, aliquots (0.5 mL) were collected from the release media and replaced with the same volume of fresh SNF. The concentration of Ola in the collected samples was determined using a previously validated HPLC method [31].

#### 2.2.4. In Vivo Pharmacokinetics and Pharmacodynamic Studies

##### Animals

Adult male Sprague-Dawley rats (8 weeks old, 200 ± 10% g) were maintained under standard laboratory conditions, including ad libitum access to food and filtered water, a controlled light/dark cycle, and regulated temperature and humidity. Animals were acclimated for one week prior to the initiation of experimental procedures. To reduce potential confounders, animals were housed in identical conditions. Treatment administration and behavioral testing were performed at the same time of day to minimize circadian variation. Cage positions were rotated weekly to avoid location-based environmental effects. All in vivo experiments were conducted in accordance with the ARRIVE guidelines, the institutional animal welfare policies of the Faculty of Pharmacy, University of Sadat City, and the international principles of laboratory animal care. The study protocol was approved by the Research Ethics Review Committee (Approval No. RERC-FOP-USC-24-02-07), and all procedures aimed to minimize animal discomfort and adhere to the principles of the 3Rs (Replacement, Reduction, Refinement). The study incorporated humane endpoints in accordance with institutional animal welfare guidelines. Animals were monitored daily for signs of pain, distress, or illness, including reduced mobility, weight loss (>15%), abnormal posture, labored breathing, and lack of grooming or food intake. If any of these signs were observed persistently or worsened, the animal would have been humanely euthanized.

##### Pharmacokinetics Study

One hundred forty-four rats were randomly assigned to three groups, with 48 animals in each group. Animals were randomly assigned using a computer-generated randomization sequence created with Microsoft Excel’s RAND function. The first and second groups received an intravenous (IV) injection or oral administration of Ola solution at a dose of 2 mg/kg body weight, respectively. The Ola solution (100 µL) in phosphate buffer saline (PBS, pH 7.4) containing DMSO (0.1% *v*/*v*) was injected via the tail vein or administered using a flexible oral gavage needle. The third group received the optimized Poly_Ola_ intranasally at the same dose of 2 mg/kg body weight. The intranasal administration (IN) was performed by installation of 10 µL Poly_Ola_ using a micropipette in each nostril. Blood samples were obtained through cardiac puncture into heparinized tubes at designated time points. Following the experimental procedures, the rats were humanely euthanized, and their brains were promptly excised. The excised brain tissues were immediately immersed in ice-cold PBS (pH 7.4) and homogenized to a final concentration of 25% *w*/*v* in PBS. Blood samples and brain homogenates were subsequently centrifuged at 9962× *g* for 15 min. The resulting plasma and supernatant from the homogenates were each mixed with an equal volume of acetonitrile to facilitate protein precipitation. After vortex mixing and centrifugation at 17,762× *g* for 10 min, the samples were stored at −80 °C for subsequent analysis. The concentrations of Ola in the plasma and brain were determined using a previously validated HPLC method [31]. The maximum plasma concentration (C_max_) and the time taken to reach C_max_ (T_max_) were calculated directly from the plasma and brain concentration-time profiles. Pharmacokinetic parameters in plasma and brain, including the area under the plasma drug curve (AUC), mean residual time (MRT), and elimination rate constant (Kel) were calculated using PK-Solver software Add-Ins for Microsoft Excel 2010 [33]. In addition, the drug-targeting efficiency percentage (DTE%) and direct-transport percentage (DTP%) were computed using the following equations.

(3) DTE%=(AUCbrainAUCplasma)IN(AUCbrainAUCplasma)IV

(4) $$DTP\%=\frac{B_{IN-}B_{X}}{B_{IN}}*100$$

where $B_{X}=\frac{B_{IV}}{P_{IV}}*P_{IN}$

B_X_ is the brain AUC_0–480min_ fraction contributed by systemic circulation, following IN administration, B_IV_ is the brain AUC_0–480min_ following IV administration, P_IV_ is the plasma AUC_0–480min_ following IV administration, B_IN_ is the brain AUC_0–480min_ following IN administration, and P_IN_ is the plasma AUC_0–480min_ following IN administration.

##### Pharmacodynamics Study

Paw placement test

The paw test was performed using a Perspex platform with dimensions of 30 × 30 × 20 cm (length × width × height). The platform featured two holes (4 cm in diameter) on the top surface for placing the forelimbs, two larger holes (5 cm in diameter) on the bottom surface for the hindlimbs, and a slit at the rear to accommodate the tail [34]. The rats were divided into three groups, each containing ten animals. The first group received an IV injection of PBS (pH 7.4, 100 µL), and the second and third groups received an IV Ola solution injection and IN Poly_Ola_ (2 mg/kg), respectively. Following 30 min of administration, the rats’ forelimbs were carefully placed into holes, followed by their hindlimbs. Forelimb retraction time (FRT) and hindlimb retraction time (HRT) were recorded as measures of sensorimotor response. FRT was defined as the duration required for the rat to retract one forelimb, whereas HRT corresponded to the time taken to retract one hindlimb. Both times were recorded with a minimum of 1 s and a maximum of 30 s [13].

Open-field tests in schizophrenia-like rat model

The open-field test was conducted to assess the locomotor activity and exploratory behavior of rats in a schizophrenia-like model induced by ketamine. Thirty rats were randomly assigned to one of three groups, each consisting of ten animals. To induce a schizophrenia-like state, all rats were administered an intraperitoneal injection of ketamine (25 mg/kg) [35]. Following this, the rats were further divided into three groups: an untreated group, a group receiving an IV injection of Ola solution (100 µL, 2 mg/kg), and a group receiving an IN Poly_Ola_ (10 µL in each nostril, 2 mg/kg) once daily for one week [13]. Each rat was positioned in the center of an open-field device of 40 × 40 × 30 cm (Accuscan Instruments, Columbus, OH, USA). The gadget was divided into 16 squares (10 × 10 cm) with black lines, where the rat’s movement was tracked. The total distance traveled was recorded to evaluate the animals’ exploratory behavior and general locomotor activity. The test was conducted 60 min after drug administration to measure both the acute effects of the treatments on ketamine-induced alterations in behavior [36]. Data from these experimental groups were compared to those from healthy, untreated control rats, which received IV injections of PBS (100 µL, pH 7.4) for 7 days, providing a baseline for normal locomotor activity and exploratory behavior.

#### 2.2.5. Comparative Effects of Oral and Intranasal Olanzapine on Metabolic and Oxidative Stress Markers in Rats

Thirty rats were randomly divided into three groups (*n* = 10 per group) and observed over eight weeks. The control animals received daily oral doses of phosphate-buffered saline (PBS, pH 7.4). The second group received oral administration of Ola solution at a dosage of 2 mg/kg/day, while the third group was treated intranasally with Poly_Ola_ at the same dose. Animal body weights were monitored every four days throughout the experiment. Once per week, the rats underwent a fasting period of 12 h before blood samples were obtained from their tail veins. Blood samples (2 mL per animal) were collected and divided into two aliquots. The first aliquot was mixed with heparin and centrifuged at 9962× *g* for min at 4 °C to obtain plasma, while the second aliquot was allowed to clot at room temperature and subsequently centrifuged to isolate serum. Both plasma and serum samples were stored at −80 °C until further analysis. Plasma glucose concentrations were assessed using a glucose colorimetric assay kit (Cell Biolabs, Inc., San Diego, CA, USA), and insulin levels were quantified with a rat insulin ELISA kit (Invitrogen, Renfrewshire, UK) using a Chro-Mate-4300 ELISA microplate reader (Awareness Technology, Inc., Palm City, FL, USA). Serum triglycerides, alanine aminotransferase (ALT), and aspartate aminotransferase (AST) levels were measured using colorimetric assay kits (Sigma-Aldrich, Dorset, UK) in accordance with the manufacturer’s protocols. At the conclusion of the study, animals were euthanized, and the testicles and epididymides were excised, rinsed with PBS (pH 7.4), and weighed. Oxidative stress biomarkers glutathione (GSH), catalase (CAT), superoxide dismutase (SOD), and malondialdehyde (MDA) were quantified using commercially available colorimetric assay kits (Sigma-Aldrich, Dorset, UK) according to the manufacturer’s instructions.

#### 2.2.6. Statistical Analysis

All in vitro experiments were repeated thrice, and the data were reported as mean ± standard deviation (SD). Prior to conducting statistical analyses, data were assessed for normality using the Shapiro-Wilk test and for homogeneity of variances using Levene’s test. Differences were considered statistically significant at *p*-values less than 0.05. In vivo pharmacokinetic and pharmacodynamic examinations’ results were reported as the mean ± standard error (SE) of six and ten replicates, respectively. The pharmacokinetic parameters were estimated with the PK-solver software. The statistical comparison Student’s *t*-test was applied to compare two variables while the ANOVA test followed by the Tukey HSD test was used for comparing different parameters between groups.

## 3. Results and Discussion

### 3.1. Preparation and Optimization of Olanzapine-Loaded Polymersomes

In this study, Poly_Ola_ was prepared using P401, a triblock copolymer that has gained attention in drug delivery due to its unique amphiphilic properties. P401, composed of poly (ethylene oxide)-block-poly (propylene oxide)-block-poly (ethylene oxide), plays a critical role in forming stable vesicular structures that enhance drug encapsulation, stability, and release control. Its ability to self-assemble in aqueous solutions makes it particularly suitable for the development of nanocarriers designed for controlled drug release [37,38]. A BBD was employed to investigate the impact of various formulation parameters, including P401 concentration, Ola concentration, and stirring speed, on the key characteristics of the formulation: particle size (Y1), EE% (Y2), and LE% (Y3). Table S1 shows the composition of the seventeen formulations prepared and the obtained responses. The relationship between the different variables and each of the measured responses was modeled using a polynomial equation. Statistical models were selected based on the highest adjusted and predicted R^2^ values, ensuring a difference of less than 0.2 between them, and the lowest prediction error sum of squares (PRESS), following the exclusion of nonsignificant variables [39]. The quadratic model was selected as the best-fit statistical model for particle size (Table S2), while the 2FI model was chosen for EE% and LE% (Tables S3 and S4). Tables S5–S7 show the ANOVA results of different variables’ effects on particle size, EE%, and LE% where *p* value less than 0.05 indicated a significant impact of the investigated parameter.

The particle size of the formulations ranged from 80.57 ± 2.5 nm to 254.65 ± 3.6 nm with EE% ranging from 76.65 ± 2.78% to 90.2 ± 3.08% and LE% ranging from 3.31 ± 0.21% to 10.38 ± 1.25%, demonstrating variability depending on the preparation conditions (Table S1). The size distribution, represented by the polydispersity index (PDI), was below 0.25 for all formulations, indicating the monodispersity of the fabricated Poly [13]. The effect of different variables on particle size, EE%, and LE% can be defined using the following equations:

Particle size (Y1) = 151.69 + 54A + 25.59B − 24.74C + 17.86AB + 10.08AC + 26.4A^2^ − 23.51B^2^ (5)

EE% (Y2) = + 82.62 − 1.17A + 3.36B − 5.07AC + 2.68BC(6)

LE% (Y3) = + 6.94 − 1.85A + 1.61B − 0.7338C + 0.8AC(7)

**Figure 1:**
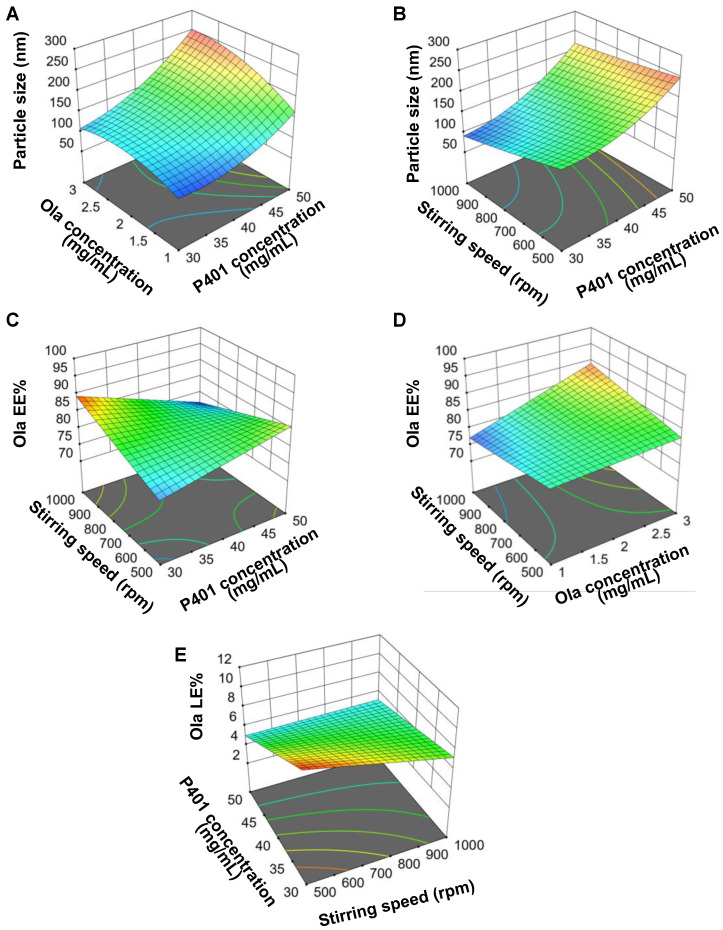
Response 3D plots for the significant parameters’ interaction on particle size, EE%, and LE% of the prepared Poly_Ola_. (**A**) The interaction of P401 concentration and Ola concentration (AB) and (**B**) of P401 concentration and stirring speed (AC) had a positive influence on particle size of Poly_Ola_. (**C**) Ola EE% is inversely proportional to the interaction of P401 concentration and stirring speed (AC). (**D**) The interaction of stirring speed and Ola concentration (BC) had a positive effect on Ola EE%. (**E**) Ola LE% is directly proportional to the interaction of P401 concentration and stirring speed (AC).

By inspecting data listed in Table S1 and Figure S1 and Figure 1, it could be deduced that the smallest particle size, 80.57 ± 2.5 nm, was observed in Run 8, where the P401 concentration was 40 mg/mL, the drug concentration was 1 mg/mL, and the stirring speed was 1000 rpm. This indicates that lower P401 and Ola concentrations, combined with high stirring speeds, result in smaller Poly (Equation (5)). Conversely, the largest particle size, 254.65 ± 3.6 nm, was found in Run 1, which had a higher P401 concentration (50 mg/mL), a higher Ola concentration (3 mg/mL), and a moderate stirring speed (750 rpm). These results suggest that higher polymer and Ola concentrations tend to produce larger particles, while increasing stirring speeds can contribute to smaller particle sizes [13]. The observed size reduction with high stirring speed is likely due to decreased viscosity and enhanced dispersion efficiency, which facilitate the formation of more uniformly distributed nanoscale particles. Moreover, from the data presented in Figure 1A,B, it can be deduced that the interaction effect of the P401 and Ola concentration (AB) or the stirring speed mixture (AC) had a positive effect on the Poly_Ola_ size.

Table S1 shows that the highest EE% of 90.2 ± 3.08% was observed in Run 12, which had a P401 concentration of 30 mg/mL, an Ola concentration of 2 mg/mL, and a stirring speed of 1000 rpm. This formulation showed the highest efficiency in drug entrapment, suggesting that lower polymer concentration, combined with moderately high drug concentration, leads to improved encapsulation. On the other hand, the lowest EE% of 76.65 ± 2.78% was recorded in Run 8 with its lowest particle size. These results indicate an inverse relationship between P401 concentration and Ola EE% as shown in Figure S2. This may be attributed to the higher viscosity of the formulation matrix at elevated polymer concentrations, which can hinder efficient drug encapsulation by limiting diffusion of the drug into the core of the Poly (Equation (6)). Conversely, increasing the Ola concentration exerted a positive effect on EE% (Figure S2, Equation (6)). This direct relationship reflects the increased availability of the drug for incorporation into the Poly structure, thereby enhancing the loading capacity of the system [13]. Additionally, a higher drug concentration may improve drug–polymer interactions, stabilizing the encapsulated drug and minimizing loss during preparation.

Figure 1C illustrates that Ola EE% is inversely related to the interaction between P401 concentration and stirring speed (AC). Figure 1D shows that the interaction of Ola concentration and stirring speed (BC) was directly proportional to Ola EE%.

The loading efficiency (LE%) ranged from 3.31 ± 0.21% to 10.38 ± 1.25% (Table S1), with the highest value found in Run 3 (P401 of 30 mg/mL, drug of 2 mg/mL, stirring speed of 500 rpm). This suggests that formulations with higher drug concentrations tended to show better LE%, as more drug was incorporated into the Poly. In contrast, the lowest LE% was observed in Run 15, where the P401 concentration was higher (50 mg/mL) and the drug concentration was lower (1 mg/mL). This outcome indicates that increasing drug concentration improved LE%, while higher polymer concentration and high stirring speed tended to lower it (Figure S3). In addition, the interaction of P401 concentration and stirring speed (AC) had a positive effect on Ola LE% (Figure 1E, Equation (7)).

### 3.2. Design Space for Optimized Poly_Ola_ Formulation

The design space was constructed by superimposing the contour plots of the studied variables to identify regions that meet the desired response outcomes. The yellow-shaded area indicates the combination of variable levels that satisfy the optimization criteria: minimizing particle size while maximizing EE% and LE% (Figure S4). The final optimized formulation, designated as Poly_Ola_, was prepared by dissolving 300 mg of P401 and 30 mg of Ola in 10 mL of PBS (pH 7.4), resulting in final concentrations of 30 mg/mL and 3 mg/mL, respectively. The preparation process followed the method described in Section 2.2.1. This specific formulation was selected as a checkpoint due to its high desirability value of 0.929. This formulation was used to validate the predictive accuracy of statistical models. Table S8 presents the expected versus experimental values for particle size, EE%, and LE%. The low percentage of predicted errors reported (Table S8) confirms the robustness and reliability of the developed models in evaluating and predicting the impact of formulation variables on achieving the desired characteristics of Poly_Ola_.

### 3.3. Characterization of the Prepared Poly_Ola_

The optimized formulation, Poly_Ola_, exhibited a particle size of 78.3 ± 4.5 nm and a PDI of 0.21 ± 0.03, indicating a monodisperse nanosystem. The formulation carried a moderately negative zeta potential (−14.64 ± 2.9 mV), suggesting colloidal stability [40,41]. Moreover, the net negative charge could be due to the presence of the PEG corona in the used polymer (P401) in Poly preparations [42]. Additionally, it demonstrated a high EE% of 91.36 ± 3.55% and LE% of 9.11 ± 1.59%, confirming the effectiveness of the encapsulation process (Table 2).

### 3.4. The Optimized Poly_Ola_ Serum Stability

**Figure 2:**
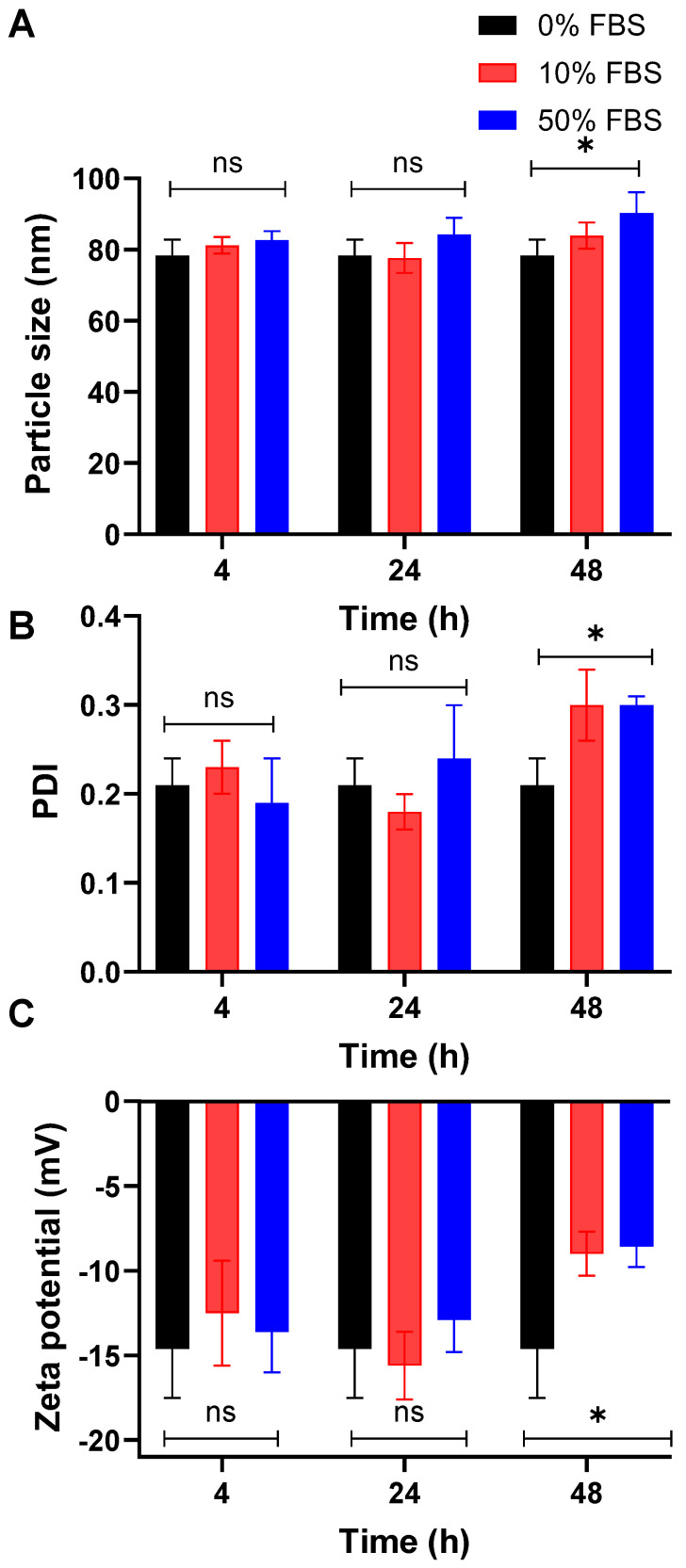
The optimized Poly_Ola_ showed improved serum stability. The optimized Poly_Ola_ is incubated with FBS (10% and 50% *v*/*v*) for 4, 24, and 48 h then (**A**) particle size, (**B**) PDI, and (**C**) zeta potential are measured using DLS. Poly_Ola_ kept its original size and zeta potential for 24 h of serum incubation. A significant change in particle size, PDI, and zeta potential is observed after 48 h of serum incubation (*p* < 0.05). Statistical analysis was performed using One-way ANOVA followed by Tukey’s post-test * *p* < 0.05, ns is nonsignificant.

To assess the colloidal stability of the optimized Poly_Ola_ formulation under biologically relevant conditions, the nanoparticles were incubated with FBS at concentrations of 10% and 50% (*v*/*v*) for 4, 24, and 48 h. The kinetic stability was evaluated by measuring particle size, polydispersity index (PDI), and zeta potential using DLS. These parameters serve as indicators of Poly_Ola_ integrity and surface interaction in serum-rich environments. At 4 and 24 h, Poly_Ola_ demonstrated stable physicochemical characteristics across all FBS concentrations, indicating effective resistance to protein-induced destabilization (Figure 2). However, by 48 h, significant changes in particle size and PDI were observed, particularly at 50% FBS (*p* < 0.05), suggesting time-dependent aggregation and loss of uniformity. Zeta potential analysis further supported these findings. Compared to the baseline zeta potential at 0% FBS (−14.6 ± 2.9 mV), exposure to 50% FBS resulted in a significant reduction in surface charge at 48 h (−8.59 ± 1.2 mV) (*p* < 0.05). This decrease in negative charge indicates a loss of electrostatic repulsion, likely due to the adsorption of serum proteins onto the Poly_Ola_ surface. These results highlight the stability of the proposed Poly_Ola_ for 24 h in the presence of biological conditions [43].

### 3.5. In Vitro Release Profile of Ola from Poly_Ola_

**Figure 3:**
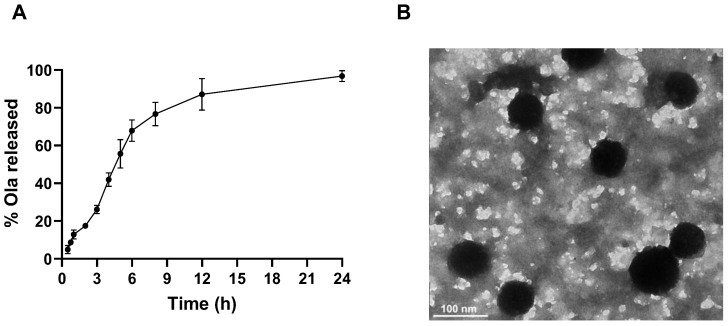
In vitro characterization of the optimized Poly_Ola_ showed controlled release. (**A**) In vitro Ola release from Poly_Ola_ in PBS (containing 0.5% *v*/*v* tween 80, pH 7.4) was measured using dialysis method at 37 °C. Ola in the dialysate is quantified by HPLC. (**B**) The Poly_Ola_ appeared as nonaggregate spherical nanostructure under transmission electron microscope with particle size in consistency with the DLS technique.

The in vitro release profile of the optimized PolyOla formulation was assessed in SNF over a 24-h period. As shown in Figure 3A, a biphasic drug release profile with an initial burst release was observed, with approximately 12.9 ± 2.32% of Ola released within the first hour. This initial phase may be attributed to the rapid diffusion of surface-associated drugs or loosely bound drug molecules at or near the Poly surface. The release continued in a sustained manner, reaching ~41.9 ± 3.57% at 4 h and ~67.9 ± 5.66% by 6 h, indicating a controlled release phase likely governed by drug diffusion through the polymeric matrix and gradual matrix relaxation or erosion [44]. By 12 h, the cumulative release reached ~87.1 ± 8.35%, ultimately achieving 96.8 ± 2.83% at 24 h, suggesting nearly complete drug liberation.

### 3.6. The Transmission Electron Micrograph of Poly_Ola_

The transmission electron microscopy (TEM) image of the optimized Poly_Ola_ formulation, presented in Figure 3B, revealed well-dispersed, non-aggregated spherical nanostructures. The observed particle size range was consistent with the hydrodynamic diameter obtained by DLS, thereby confirming the uniformity and nanoscale dimensions of the formulation. The spherical morphology and nano-sized characteristics of Poly_Ola_ are anticipated to facilitate enhanced mucosal permeability and prolonged retention at the nasal epithelium, which are critical factors for improving drug absorption and therapeutic efficacy in intranasal delivery systems [13].

### 3.7. Pharmacokinetics Profile of Ola from the Prepared Nasal Poly_Ola_ Formulation

**Figure 4:**
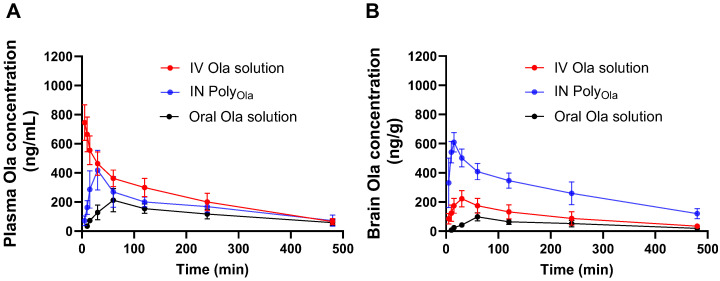
Olanzapine pharmacokinetic profile in rats’ (**A**) plasma and (**B**) brain after administration of IN Poly_Ola_, IV Ola solution, and oral Ola solution. Animals received a dose of 2 mg/kg of Ola either as a solution via IV injection through the tail vein, oral administration, or IN Poly_Ola_. At each time point, 6 animals were sacrificed from each group and the concentration of Ola in plasma and brain was quantified using HPLC. A significantly higher brain Ola concentration was observed at all time points than in IV and oral solution. Data points represent the mean ± SE (*n* = 6).

To investigate the pharmacokinetic performance and brain-targeting efficiency of the developed Poly_Ola_ formulation, Ola concentrations in rat plasma and brain were determined following IN administration of Poly_Ola_ and compared to IV and oral Ola solutions. Figure 4A,B present the time–concentration profiles and Table 3 summarizes the pharmacokinetic parameters, DTE%, and direct transport DTP%. As shown in Figure 4A, IV administration of Ola resulted in the highest initial plasma concentrations due to immediate systemic availability. However, IN administration of Poly_Ola_ achieved a significantly higher C_max_ (418.21 ± 45.69 ng/mL) compared to the oral solution (212.38 ± 24.36 ng/mL), with a T_max_ of 30 min for Poly_Ola_ and 60 min for oral Ola. The AUC_0–480min_ for IN Poly_Ola_ (82.53 ± 8.14 µg·min/mL) was also substantially greater than that of the oral route (55.34 ± 6.12 µg·min/mL), indicating improved systemic bioavailability via the nasal pathway. These findings suggest that the nasal route enabled rapid absorption, likely due to the bypassing of first-pass hepatic metabolism and facilitated permeation across the nasal mucosa. The MRT for IN Poly_Ola_ (2.96 ± 0.34 h) was comparable to that of the IV formulation (2.62 ± 0.27 h) and oral solution (3.24 ± 0.39 h), demonstrating the ability of the nanocarrier to maintain plasma levels over time (*p* > 0.05). The observed K_el_ was slightly lower for IN Poly_Ola_ (0.17 ± 0.02 h^−1^) compared to IV (0.25 ± 0.03 h^−1^) and oral (0.16 ± 0.02 h^−1^), indicating a sustained drug presence in circulation (*p* < 0.05).

The brain pharmacokinetic profile of IN Poly_Ola_ (Figure 4B) further confirmed its superior targeting potential. The C_max_ in brain tissues reached 609.46 ± 65.98 ng/mL, which was markedly higher than both IV (222.65 ± 25.32 ng/mL) and oral (98.21 ± 12.11 ng/mL) administrations. Importantly, IN Poly_Ola_ achieved this peak at only 15 min, compared to 30 and 60 min for IV and oral routes, respectively, indicating rapid brain uptake through the olfactory and trigeminal neural pathways. Additionally, IN Poly_Ola_ exhibited the highest brain AUC_0–480min_ (131.68 ± 11.96 µg·min/mL), nearly 2.8-fold and 5.7-fold greater than IV and oral formulations, respectively. The MRT of 2.96 ± 0.32 h and reduced K_el_ of 0.17 ± 0.01 h^−1^ suggest extended drug retention in the brain, enhancing the duration of the pharmacological effect. This enhanced targeting efficiency is quantitatively supported by a DTE% of 365.38% and a DTP% of 72.63% (Table 3). These values confirm the significant contribution of direct nose-to-brain transport in bypassing systemic circulation, thereby reducing peripheral exposure and enhancing central nervous system delivery. The superior pharmacokinetic behavior of IN Poly_Ola_ can be attributed to multiple synergistic features. The nasal cavity provides a unique anatomical and physiological interface with the CNS, enabling the direct transport of therapeutic agents via the olfactory epithelium and trigeminal nerve pathways, bypassing the blood–brain barrier and hepatic first-pass metabolism. The hydrophobic core of the Poly encapsulates Ola efficiently, enhancing solubility and protecting the drug from enzymatic degradation. The sub-100 nm particle size not only enhances the surface area for absorption but also facilitates rapid translocation through the olfactory epithelium and fila olfactoria, enabling direct CNS entry via axonal transport [45]. Moreover, the inclusion of Pluronic^®^ block copolymers in the formulation may contribute to enhanced brain penetration by inhibiting P-glycoprotein-mediated efflux and facilitating transcytosis across the BBB [46]. These characteristics combined with Poly_Ola_ flexibility, mucoadhesive behavior, and sustained-release kinetics underscore its potential as a non-invasive and highly efficient nanocarrier platform for CNS drug delivery. This multifaceted transport mechanism underpins the rapid brain accumulation and prolonged retention of Ola following IN delivery.

Several nanocarrier systems have been previously developed to enhance the IN delivery of Ola, including PLGA nanoparticles, chitosan-based systems, polymeric micelles, hydrophobized starch nanoparticles, and transfersomal vesicles. For instance, PLGA nanoparticles achieved a brain AUC increase of 6.35-fold over IV and 10.86-fold over IN solution, with 68.91% entrapment efficiency and a particle size of ~91 nm [16]. Chitosan nanoparticles delivered via the IN route exhibited 51% absolute bioavailability with a size of ~208 nm and ~87% encapsulation [47]. Transfersomal vesicles, while achieving a high brain AUC of 36,486.3 ng·min/mL, had a larger and less consistent particle size range (310–885 nm), which may limit mucosal penetration [48]. More recently, polymeric micelles developed by our group with a very small size (~39.25 nm) and 28.15% entrapment efficiency demonstrated a 4.1-fold increase in brain AUC_0–480min_ versus IV solution [13]. In comparison, Poly_Ola_ showed superior characteristics, achieving a high entrapment efficiency and an optimal particle size, resulting in a 5.7-fold increase in brain AUC versus oral Ola and a DTE% of 365%. These findings collectively demonstrate that the Poly_Ola_ formulation exhibits comparable or superior performance to other IN Ola delivery systems in terms of EE%, brain targeting efficiency, serum stability, and translational feasibility.

While the pharmacokinetic results demonstrated significantly enhanced brain uptake of Ola following IN administration of Poly_Ola_ evidenced by rapid T_max_, high brain C_max_, elevated AUC, and DTE%, the proposed mechanism of direct nose-to-brain transport remains inferential. These findings are consistent with known advantages of the IN route for CNS drug delivery, particularly through the olfactory and trigeminal pathways. However, the current study did not include direct mechanistic evaluations such as biodistribution imaging, radiolabeling, or fluorescent tracking of the nanocarrier to visually confirm the route of transport. This represents a key limitation, and future investigations are warranted to incorporate such techniques to quantitatively distinguish between direct nose-to-brain trafficking and systemic redistribution. Despite this, the pharmacokinetic profile and rapid onset of brain action observed strongly support the likelihood of neural pathway involvement in the enhanced delivery performance of Poly_Ola_.

### 3.8. Pharmacodynamic Assessment of Poly_Ola_ in Schizophrenia’s Model Rats

**Figure 5:**
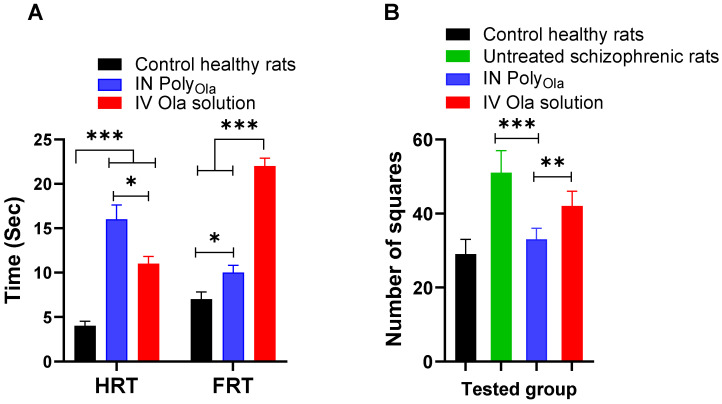
Assessment of pharmacodynamic effect of IV Ola solution and IN Poly_Ola_. (**A**) Paw test, (**B**) in ketamine-induced schizophrenia in rats by open-field test and (**B**). A significantly higher anti-schizophrenic effect and fewer extrapyramidal side effects were observed following IN administration of Poly_Ola_ compared to IV solution. Data points represent the mean ± SE (*n* = 10). ANOVA was used to compare different parameters between groups, followed by the Tukey HSD test, * *p* < 0.05, ** *p* < 0.01, and *** *p* < 0.001.

To assess the neurobehavioral safety and potential central nervous system effects of the administered IN Poly_Ola_, the FRT, and HRT were measured in rats following treatment. These reflex response tests serve as indicators of sensorimotor coordination and CNS functionality. In the healthy control group, baseline FRT and HRT values were within the normal physiological range [49], indicating intact reflex responses (Figure 5A). Rats treated with IV Ola solution exhibited a significant prolongation in both FRT and HRT (*p* < 0.001), reflecting a sedative or suppressive effect on CNS activity, likely due to systemic distribution and rapid brain penetration of the drug [13]. Conversely, rats receiving IN Poly_Ola_ displayed a significantly increased HRT compared to controls, suggesting effective brain targeting and prolonged central activity (*p* < 0.001). However, the FRT in this group showed remarkably lower levels, compared to the IV group which may indicate a more favorable balance between therapeutic CNS engagement and motor coordination preservation (Figure 5A). These findings support the neurobehavioral safety of IN Poly_Ola_, highlighting its ability to enhance brain delivery of Ola without inducing excessive systemic sedation or compromising motor reflexes. These findings are consistent with our previous study on IN Ola-loaded polymeric micelles, which demonstrated that nasal administration can enhance both the efficacy and safety profile of Ola [13].

To assess the antipsychotic efficacy of the developed IN Poly_Ola_ formulation, a behavioral pharmacodynamic study was conducted in an animal model of schizophrenia. The therapeutic outcomes were evaluated by comparing treatment groups against both healthy control and untreated schizophrenic rats. The measured parameter presumably latency or behavioral score is indicative of antipsychotic effectiveness, where lower values represent improved cognitive and behavioral performance [35]. Figure 5B shows that the untreated schizophrenic group exhibited a significant behavioral deficit (51 ± 2.45 squares), confirming the successful induction of schizophrenia-like symptoms. In contrast, healthy control animals maintained normal behavior, with a value of 29 ± 1.63 squares. Treatment with IV Ola solution produced a noticeable improvement (42 ± 1.63 squares), but the effect remained suboptimal relative to the healthy baseline (*p* < 0.05). Remarkably, IN administration of the Poly_Ola_ formulation resulted in a greater behavioral restoration, yielding a value of 33 ± 1.22 squares, which was statistically closer to the healthy control group than the IV-treated group (*p* > 0.05). This indicates that IN Poly_Ola_ offers a more effective therapeutic response, likely due to enhanced brain targeting and rapid onset of action via direct nose-to-brain transport. These findings are in strong agreement with the pharmacokinetic and neurobehavioral data, where IN Poly_Ola_ demonstrated higher brain concentrations, faster T_max_, and improved safety profiles. The superior pharmacodynamic performance further reinforces the hypothesis that small particle size (<100 nm), bypass of hepatic first-pass metabolism, and targeted delivery via the olfactory route significantly enhance the therapeutic efficacy of Ola [50].

It is important to acknowledge a limitation in the present study regarding the absence of IV and oral administration of the PolyOla formulation as comparator groups. While the pharmacokinetic and pharmacodynamic evaluations demonstrated significantly improved brain targeting and therapeutic efficacy of IN PolyOla compared to conventional IV and oral Ola solutions, the design does not fully isolate the effect of the IN route from the potential advantages conferred by the Poly nanocarrier itself. Including IV and oral PolyOla arms would have allowed for a more definitive assessment of the role of the administration route versus formulation properties in enhancing drug delivery to the brain. Future studies are therefore warranted to include these additional comparator groups, which would enable a clearer understanding of whether the observed enhancements are primarily attributable to the nose-to-brain pathway, the physicochemical characteristics of the Poly, or a synergistic combination of both. Despite this limitation, the current findings provide compelling evidence for the IN route as an effective and non-invasive strategy for CNS drug delivery, particularly when combined with nanoscale carriers designed for mucosal permeation and brain penetration.

### 3.9. Evaluation of Metabolic and Hepatic Safety Profiles

**Figure 6:**
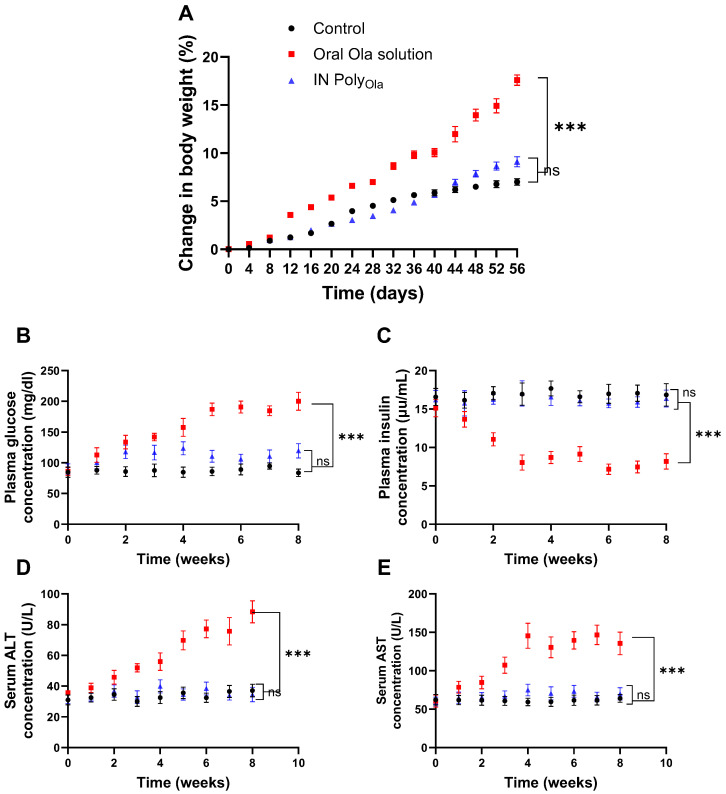
Evaluation of metabolic and hepatic safety following oral administration of oral Ola solution and IN Poly_Ola_ in rats. (**A**) Percentage change in body weight, (**B**) plasma glucose levels, (**C**) plasma insulin levels, and hepatic enzyme levels (**D**) ALT, and (**E**) AST in rats over 8 weeks following treatment with oral Ola solution or IN Poly_Ola_. Data are presented as mean ± SE (*n* = 10 per group). Significant differences were assessed using one-way ANOVA followed by Tukey’s post hoc test, *** *p* < 0.001 and ns refers to nonsignificant.

To comprehensively assess the metabolic side effects associated with Ola delivery via the IN Poly_Ola_, changes in body weight were monitored over a 56-day period. As illustrated in Figure 6A, rats administered oral Ola solution exhibited a progressive and pronounced increase in body weight, reaching 17.59 ± 1.69% by day 56. This significant weight gain aligns with the well-documented metabolic adverse effects of systemic Ola, which include appetite stimulation, altered glucose homeostasis, and fat deposition [51,52]. In contrast, animals receiving IN Poly_Ola_ displayed a substantially attenuated body weight gain of 9.11 ± 1.65%, which was statistically lower than that of the oral group (*p* < 0.001). The control group showed only a modest, physiologically normal increase of 7.00 ± 1.06%, which did not differ significantly from the IN Poly_Ola_ group (*p* > 0.05). These findings suggest that IN administration of Ola via Poly_Ola_ may circumvent the metabolic disturbances typically induced by systemic exposure, offering a more favorable profile for long-term treatment.

In parallel, plasma glucose and insulin concentrations were measured weekly for eight weeks to further evaluate glycemic control (Figure 6B,C). The oral Ola group demonstrated a marked hyperglycemic response, with plasma glucose levels rising to 200.05 ± 14.65 mg/dL, accompanied by a significant reduction in insulin secretion (8.18 ± 1.01 μIU/mL). These alterations are consistent with Ola’s established diabetogenic effects, driven by insulin resistance and β-cell dysfunction [53]. Strikingly, rats treated with IN Poly_Ola_ maintained near-normoglycemic plasma glucose levels (119.58 ± 11.69 mg/dL) and preserved insulin levels (16.36 ± 1.10 μIU/mL). Both parameters were significantly different from the oral group (*p* < 0.001), yet statistically comparable to the control group (*p* > 0.05). These outcomes highlight the ability of IN Poly_Ola_ to protect against Ola-induced metabolic dysregulation, likely due to reduced systemic exposure and enhanced brain-targeted delivery that spares peripheral tissues from off-target effects such as hyperglycemia and insulin suppression.

To further investigate organ-specific safety, the study also examined markers of hepatic function, namely serum ALT and AST, over the 8-week period Figure 6D,E. The oral Ola group showed significant hepatocellular stress, with ALT and AST levels elevated to 88.39 ± 7.18 U/L and 135.64 ± 14.78 U/L, respectively [54]. These levels were significantly higher than those observed in both the IN Poly_Ola_ and control groups (*p* < 0.001), indicating potential hepatotoxic effects linked to systemic drug distribution and hepatic metabolism. Conversely, the IN Poly_Ola_ group maintained hepatic enzyme levels within the physiological range, with ALT at 34.56 ± 4.67 U/L and AST at 69.89 ± 7.98 U/L, showing no statistical difference from the healthy controls (*p* > 0.05). These findings underscore the hepatoprotective advantage of the IN route, which bypasses first-pass hepatic metabolism, thereby minimizing hepatic drug burden and associated toxicity.

Taken together, the results provide compelling evidence that IN Poly_Ola_ offers remarkably improved safety over oral olanzapine, with reduced impacts on body weight, glucose-insulin balance, and liver function. These benefits, coupled with the demonstrated efficacy of nose-to-brain delivery, support the potential of IN Poly_Ola_ as a superior therapeutic strategy for chronic CNS disorders, particularly in patients at risk for metabolic syndrome or liver dysfunction.

### 3.10. Evaluation of Oxidative Stress Markers in Testicular Tissue

**Figure 7:**
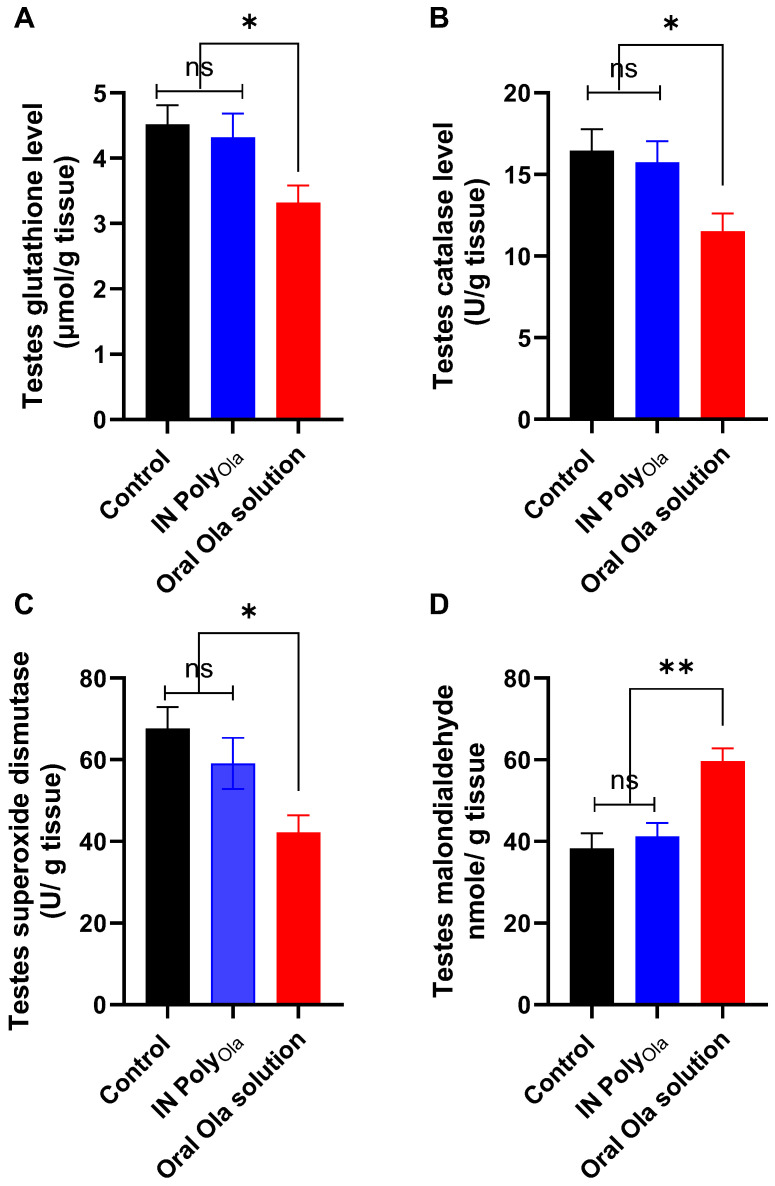
Evaluation of oxidative stress markers in testicular tissue following oral administration of oral Ola solution and IN Poly_Ola_ in rats. (**A**) Glutathione level, (**B**) Catalase activity, (**C**) superoxide dismutase, and (**D**) malondialdehyde in rats’ testes over 8 weeks following treatment with oral Ola solution or IN Poly_Ola_. Data are presented as mean ± SE (*n* = 10 per group). Significant differences were assessed using one-way ANOVA followed by Tukey’s post hoc test, * *p* < 0.05, ** *p* < 0.01, and ns refers to nonsignificant.

To investigate the oxidative stress-inducing potential of Ola and evaluate the protective effect of the IN Poly_Ola_ formulation on testicular redox status, several key biomarkers were assessed, including reduced GSH, CAT, SOD, and MDA (Figure 7A–D). In the oral Ola-treated group, GSH levels significantly declined to 3.32 ± 0.26 µmol/g compared to 4.52 ± 0.29 µmol/g in the control group (*p* < 0.05), indicating depletion of intracellular thiol reserves and heightened oxidative burden. Conversely, rats treated with IN Poly_Ola_ maintained GSH concentrations at 4.32 ± 0.36 µmol/g, statistically indistinguishable from the control group (*p* > 0.05), reflecting the formulation’s ability to preserve non-enzymatic antioxidant capacity. Similarly, CAT activity, a crucial enzymatic defense against hydrogen peroxide, was significantly reduced in the oral Ola group (11.53 ± 1.08 U/mg protein) compared to controls (16.45 ± 1.33 U/mg, *p*< 0.05), whereas IN Poly_Ola_ preserved catalase activity at 15.74 ± 1.29 U/mg (*p* > 0.05 vs. control), suggesting sustained enzymatic detoxification capacity. SOD activity, another critical component of the antioxidant defense system, was markedly suppressed in the oral Ola group (42.25 ± 4.11 U/mg) relative to controls (67.65 ± 5.22 U/mg, *p* < 0.05), indicative of superoxide accumulation and impaired redox homeostasis. Notably, IN Poly_Ola_-treated rats exhibited a partial restoration of SOD activity (59.11 ± 6.25 U/mg), significantly higher than the oral group (*p* < 0.05) and approaching control levels, further supporting the antioxidative potential of the nasal delivery route. In terms of lipid peroxidation, MDA levels were highest in the oral Ola group (59.63 ± 3.22 nmol/g), reflecting severe oxidative damage to testicular membranes, while IN Poly_Ola_ significantly attenuated MDA accumulation (41.25 ± 3.28 nmol/g), comparable to that of the control group (38.33 ± 3.66 nmol/g). These findings collectively demonstrate that oral Ola induces considerable oxidative stress in testicular tissue, as evidenced by impaired enzymatic and non-enzymatic antioxidant defenses and elevated lipid peroxidation [55]. In contrast, IN administration of Ola via the Poly_Ola_ nanocarrier effectively preserved testicular antioxidant balance and mitigated oxidative injury, highlighting its potential as a safer and more targeted therapeutic strategy for reducing drug-induced reproductive toxicity.

From a translational perspective, the use of biocompatible materials, scalable fabrication conditions, and non-invasive administration routes make PolyOla a promising platform for clinical development. Its ability to deliver Ola directly to the brain while minimizing systemic exposure may help mitigate common side effects associated with oral therapy, such as metabolic disturbances and hepatic toxicity. This could be particularly beneficial for chronic conditions like schizophrenia, where patient compliance and tolerability are critical for long-term treatment success. Despite the promising outcomes demonstrated by the intranasal Poly_Ola_ formulation in enhancing brain delivery and antipsychotic efficacy, some limitations of the study should be acknowledged. First, while the ketamine-induced schizophrenia model is widely used, it does not fully capture the complexity of human schizophrenia, particularly with respect to negative and cognitive symptoms, which may limit translational relevance. Another limitation lies in the inference of nose-to-brain delivery; although pharmacokinetic data support this mechanism, the absence of direct biodistribution imaging or fluorescent tracking restricts definitive confirmation. Moreover, sample size determination was based on prior studies rather than a formal a priori power analysis, which may affect the precision of the reported effect sizes. Addressing these limitations in future work through imaging studies, expanded behavioral models, and rigorous statistical planning will further strengthen the translational potential of this delivery platform.

## 4. Conclusions

This study successfully developed and optimized a novel IN Poly_Ola_ for the targeted delivery of Ola to the brain. The formulation demonstrated optimized physicochemical characteristics, including nanoscale size, high entrapment efficiency, and robust colloidal stability. In vitro studies confirmed sustained drug release and resistance to serum-induced destabilization, while in vivo pharmacokinetic analyses revealed significantly enhanced brain bioavailability, rapid onset of action, and prolonged residence time following IN administration. Pharmacodynamic evaluations further corroborated the superior antipsychotic efficacy and reduced extrapyramidal side effects of IN Poly_Ola_ compared to conventional IV and oral formulations. Importantly, IN Poly_Ola_ also exhibited a markedly improved safety profile by minimizing olanzapine-induced metabolic disturbances, hepatic stress, and oxidative damage in reproductive tissues. These findings collectively highlight the potential of Poly_Ola_ as a non-invasive, efficient, and organ-sparing strategy for central nervous system drug delivery. The integration of polymersome technology with IN administration offers a promising platform for enhancing the therapeutic index of antipsychotic agents, with broad implications for the future treatment of schizophrenia and other neuropsychiatric disorders.

## Figures and Tables

**Table 1:** Independent variables and corresponding responses in the Box–Behnken design applied for the optimization of olanzapine-loaded polymersome formulations.

Factors	Levels	
	Low	High
A: P401 concentration (mg/mL) ^a^	30	50
B: Ola concentration (mg/mL) ^b^	1	3
C: Stirring speed (rpm)	500	1000
Responses	Constraints	
Y1: Particle size (nm)	Minimize	
Y2: EE (%)	Maximize	
Y3: LE (%)	Maximize	

^a^ P401 is Poloxamer 401. ^b^ Ola is olanzapine.

**Table 2:** In vitro characterization of the optimized polymersomes (Poly_Ola_) ^a^.

Formula	Particle Size (nm) ^b,f^	PDI ^b,f^	Zeta Potential (mV) ^c,f^	EE% ^d,f^	LE% ^e,f^
Poly_Ola_	78.3 ± 4.5	0.21 ± 0.03	−14.64 ± 2.9	91.36 ± 3.55	9.11 ± 1.59

^a^ Poly_Ola_ is composed of ploxamer 401 (30 mg/mL) and olanzapine (3 mg/mL) stirred at 850 rpm. ^b^ measured by dynamic light scattering technique after dilution in deionized water (1:100 *v*/*v*). ^c^ measured by electrophoresis technique after dilution in deionized water (1:100 *v*/*v*). ^d^ calculated directly as percentage of olanzapine added, determined by HPLC. ^e^ calculates the percentage of entrapped olanzapine weight to total polymersome weight. ^f^ expressed as mean ± SD.

**Table 3:** Pharmacokinetic parameters of olanzapine in rat plasma and brain.

Parameter	Plasma			Brain		
	IV Ola Solution	Oral Ola Solution	IN Poly_Ola_	IV Ola Solution	Oral Ola Solution	IN Poly_Ola_
C_max_ (ng/mL)	-	212.38 ± 24.36	418.21 ± 45.69	222.65 ± 25.32	98.21 ± 12.11	609.46 ± 65.98
T_max_ (min)	-	60	30	30	60	15
AUC _0–480 min_ (µg/mL.h)	108.34 ± 9.69	55.34 ± 6.12	82.53 ± 8.14	47.31± 7.33	23.21 ± 3.55	131.68 ± 11.96
MRT (h)	2.62 ± 0.27	3.24 ± 0.39	2.96 ± 0.34	2.766±	3.15±	2.96±
Kel (h^−1^)	0.25 ± 0.03	0.16 ± 0.02	0.17 ± 0.02	0.28±	0.2±	0.17±
DTE (%)	-	-	-	-	-	365.38
DTP (%)	-	-	-	-	-	72.63

## Data Availability

The original contributions presented in this study are included in the article/Supplementary Materials. Further inquiries can be directed to the corresponding authors: Ahmed A. Katamesh and Hend Mohamed Abdel-Bar.

## Supplementary Materials

The following supporting information can be downloaded at: https://www.mdpi.com/article/10.3390/pharmaceutics17070811/s1, Table S1. Physicochemical characterization of olanzapine loaded polymersomes in the designed formulations. Table S2. Model summary statistics for particle size (Y1). Table S3. Model summary statistics for EE% (Y2). Table S4. Model summary statistics for LE% (Y3). Table S5. ANOVA of the obtained data from Box-Behnken design for the particle size of olanzapine loaded polymersomes and associated *p*-values. Table S6. ANOVA of the obtained data from Box-Behnken design for the entrapment efficiency % of olanzapine loaded polymersomes and associated *p*-values. Table S7. ANOVA of the obtained data from Box-Behnken design for the loading efficiency % of olanzapine loaded polymersomes and associated p-values. Table S8. The experimental and predicted physicochemical characterization of the optimized polymersomes (Poly_Ola_) ^a^. Figure S1. The effect of different significant variables on olanzapine loaded polymersomes particle size (Y1). (A) P401 concentration and (B) Ola concentration had a positive influence on particle size. (C) Poly_Ola_ particle size is inversely proportional to stirring speed. Figure S2. The effect of different significant variables on olanzapine loaded polymersomes EE% (Y2). (A) Increasing P401 concentration decreases EE%. (B) Ola concentration has a positive effect on EE%. Figure S3. The effect of different significant variables on olanzapine loaded polymersomes LE% (Y3). (A) increasing P401 concentration decreased the LE%. (B) Ola concentration had a positive influence on LE%. (C) Poly_Ola_ LE% is inversely proportional to stirring speed. Figure S4. Overlay plots depicting the design space region for the optimized Poly_Ola_. The design space was plotted by overlapping variables contour plots to obtain required responses. The yellow area represents the values of variables when optimized to fulfill optimization criteria; minimum particle size, maximum EE % and LE%.
