# Supplementary material for: Tunable Intranasal Polymersome Nanocarriers Triggered Olanzapine Brain Delivery and Improved In Vivo Antipsychotic Activity
Source: Pharmaceutics. 2025 Jun 23;17(7):811. doi: 10.3390/pharmaceutics17070811 (PMC12297938; doi:10.3390/pharmaceutics17070811)
Supplement: Supplementary file 1 [file pharmaceutics-17-00811-s001.zip › pharmaceutics-3632924-supplementary.pdf]

Tunable intranasal polymersome nanocarriers triggered olanzapine brain delivery and improved in vivo antipsychotic activity

Ahmed A. Katamesh [1*], Hend Mohamed Abdel-Bar [2*], Rania Mahafdeh [3], Mohammed Khaled Bin Break [4,5], Shimaa M. Hassoun [6], Gehad M. Subaiea [6], Mostafa E. El-Naggar [7], Khaled Almansour [1], Hadel A. Abo El-Enin [8], Heba A Yassin [9]

[1] Department of Pharmaceutics, College of Pharmacy, University of Ha'il, Ha'il 81442, Saudi Arabia (a.katamsh@uoh.edu.sa).

[2] Department of Pharmaceutics, Faculty of Pharmacy, University of Sadat City, Menoufia 32897, Egypt (hend.abdelbar@fop.usc.edu.eg).

[3] Department of clinical pharmacy and therapeutics, Faculty of Pharmacy, Jadara University, Irbid 21110, Jordan.

[4] Department of Pharmaceutical Chemistry, College of Pharmacy, University of Ha'il, Ha'il 81442, Saudi Arabia.

[5] Medical and Diagnostic Research Centre, University of Ha'il, Ha'il 55473, Saudi Arabia.

[6] Department of Pharmacology, College of Pharmacy, University of Ha'il, Ha'il 81442, Saudi Arabia.

[7] Department of Pharmacology and Toxicology, Faculty of Pharmacy, University of Sadat City, Menoufia 32897, Egypt.

[8] Department of Pharmaceutics, Egyptian Drug Authority, Giza 12511, Egypt.

[9] Department of Pharmaceutics and Pharmaceutical Technology, Faculty of Pharmacy, Pharos University in Alexandria, Alexandria 21648, Egypt.

* *Correspondence:* (hend.abdelbar@fop.usc.edu.eg, a.katamsh@uoh.edu.sa).

Table S1. Physicochemical characterization of olanzapine loaded polymersomes in the designed formulations.

Run	A: P401 concentration (mg/mL)	B: Ola concentration (mg/mL)	C: Stirring speed (rpm)	Particle size (nm) [a, d]	EE (%) [b, d]	LE (%) [c, d]
1	50	3	750	254.65± 3.6	84.86± 3.25	6.72± 0.84
2	50	2	1000	220.31± 5.2	77.43± 2.58	5.28± 0.36
3	30	2	500	160.21± 4.2	78.38± 4.65	10.38±1.25
4	40	3	1000	130.55± 3.6	89.07± 1.85	7.78± 1.01
5	40	2	750	154.64± 4.5	83.68± 4.14	5.73± 0.67
6	50	2	500	249.66± 3.9	85.87± 2.95	5.08± 0.74
7	40	3	500	174.36± 4.5	83.34± 4.11	9.58± 1.54
8	40	1	1000	80.57± 2.5	76.65± 2.78	4.55± 0.28
9	40	2	750	150.69± 4.2	80.25± 1.95	6.94± 0.74
10	40	2	750	145.61± 1.8	82.85± 4.51	7.38± 0.98
11	40	2	750	153.23± 3.7	80.69± 2.41	7.68± 0.36
12	30	2	1000	90.55± 4.5	90.2± 3.08	7.38± 0.48
13	40	2	750	154.27± 1.9	83.15± 3.5	6.98± 1.01
14	30	3	750	112.52± 4.7	87.16± 1.45	9.96± 0.68
15	50	1	750	160.91± 2.8	78.75± 2.65	3.31± 0.21
16	40	1	500	135.63± 3.1	81.64± 4.07	5.82± 0.47
17	30	1	750	90.24± 5.9	80.5± 3.55	7.45± 0.98

[a] measured by dynamic light scattering technique after dilution in deionized water (1: 100 v/v).
[b] calculated directly as percentage of Ola added, determined by HPLC.
[c] calculated as percentage of entrapped Ola weight to total Poly weight.
[d] expressed as mean ±SD.

Table S2. Model summary statistics for particle size (Y1).

Source	Std. Dev.	R^2	Adjusted R^2	Predicted R^2	PRESS	
Linear	23.02	0.8293	0.7899	0.6446	14341.69	
2FI	22.75	0.8717	0.7948	0.3493	26255.50	
Quadratic [a]	4.69	0.9962	0.9913	0.9588	1660.91	Suggested
Cubic	3.73	0.9986	0.9945		*	Aliased

[a] Adequate precision equals 48.61 and coefficient of variation (C.V.) % is 3.05.
* Case(s) with leverage of 1.0000: PRESS statistic not defined.

Table S3. Model summary statistics for EE% (Y2).

Source	Std. Dev.	R^2	Adjusted R^2	Predicted R^2	PRESS	
Linear	3.33	0.4176	0.2832	-0.1771	291.38	
2FI [a]	1.13	0.9485	0.9176	0.9100	22.27	Suggested
Quadratic	1.25	0.9561	0.8998	0.8563	35.56	
Cubic	1.55	0.9613	0.8454		*	Aliased

[a] Adequate precision equals17.2 and coefficient of variation (C.V.) % is 1.37.
* Case(s) with leverage of 1.0000: PRESS statistic not defined.

Table S4. Model summary statistics for LE% (Y3).

Source	Std. Dev.	R^2	Adjusted R^2	Predicted R^2	PRESS	
Linear	0.6355	0.9090	0.8880	0.8464	8.86	
2FI [a]	0.4917	0.9581	0.9330	0.9381	3.57	Suggested
Quadratic	0.5805	0.9591	0.9065	0.8980	5.88	
Cubic	0.7428	0.9618	0.8470		*	Aliased

[a] Adequate precision equals 22.4 and coefficient of variation (C.V.) % is 7.08.
* Case(s) with leverage of 1.0000: PRESS statistic not defined.

Table S5. ANOVA of the obtained data from Box-Behnken design for the particle size of olanzapine loaded polymersomes and associated p-values.

Source	Sum of Squares	Df [a]	Mean Square	F-value	p-value [b]
Model	40198.61	9	4466.51	202.94	< 0.0001*
A-P401 concentration	23329.08	1	23329.08	1059.97	< 0.0001*
B-Ola concentration	5239.30	1	5239.30	238.05	< 0.0001*
C-Stirring speed	4894.56	1	4894.56	222.39	< 0.0001*
AB	1276.63	1	1276.63	58.00	0.0001*
AC	406.22	1	406.22	18.46	0.0036*
BC	31.64	1	31.64	1.43	0.2695
A^2	2934.23	1	2934.23	133.32	< 0.0001*
B^2	2326.55	1	2326.55	105.71	< 0.0001*
C^2	18.50	1	18.50	0.8405	0.3898
Residual	154.06	7	22.01		
Lack of Fit	98.37	3	32.79	2.35	0.2132
Pure Error	55.70	4	13.92		
Cor Total	40352.67	16			

[a] Degree of freedom
[b] *$p < 0.05$

Table S6. ANOVA of the obtained data from Box-Behnken design for the entrapment efficiency % of olanzapine loaded polymersomes and associated p-values

Source	Sum of Squares	Df [a]	Mean Square	F-value	p-value [b]
Model	234.81	6	39.13	30.71	< 0.0001*
A-P401 concentration	10.88	1	10.88	8.54	0.0153*
B-Ola concentration	90.38	1	90.38	70.92	< 0.0001*
C-Stirring speed	2.12	1	2.12	1.66	0.2259
AB	0.075	1	0.075	0.059	0.8125
AC	102.62	1	102.62	80.51	< 0.0001*
BC	28.73	1	28.73	22.54	0.0008*
Residual	12.75	10	1.27		
Lack of Fit	3.18	6	0.5293	0.2213	0.9497
Pure Error	9.57	4	2.39		
Cor Total	247.55	16			

[a] Degree of freedom
[b] *$p < 0.05$

Table S7. ANOVA of the obtained data from Box-Behnken design for the loading efficiency % of olanzapine loaded polymersomes and associated p-values

Source	Sum of Squares	Df [a]	Mean Square	F-value	p-value [b]
Model	55.28	6	9.21	38.11	< 0.0001*
A-P401 concentration	27.31	1	27.31	112.96	< 0.0001*
B-Ola concentration	20.83	1	20.83	86.18	< 0.0001*
C-Stirring speed	4.31	1	4.31	17.82	0.0018*
AB	0.2025	1	0.2025	0.8377	0.3816
AC	2.56	1	2.56	10.59	0.0087*
BC	0.0702	1	0.0702	0.2905	0.6017
Residual	2.42	10	0.2417		
Lack of Fit	0.2105	6	0.0351	0.0636	0.9975
Pure Error	2.21	4	0.5517		
Cor Total	57.70	16			

[a] Degree of freedom
[b] *p< 0.05

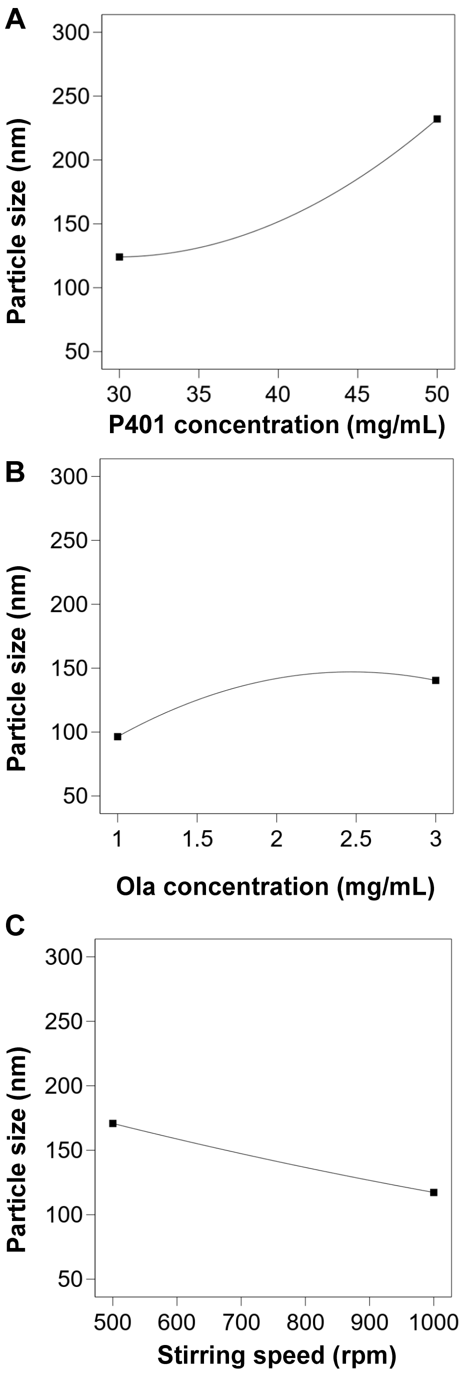

Figure S1. The effect of different significant variables on olanzapine loaded polymersomes particle size (Y1). (A) P401 concentration and (B) Ola concentration had a positive influence on particle size. (C) $Poly_{Ola}$ particle size is inversely proportional to stirring speed.

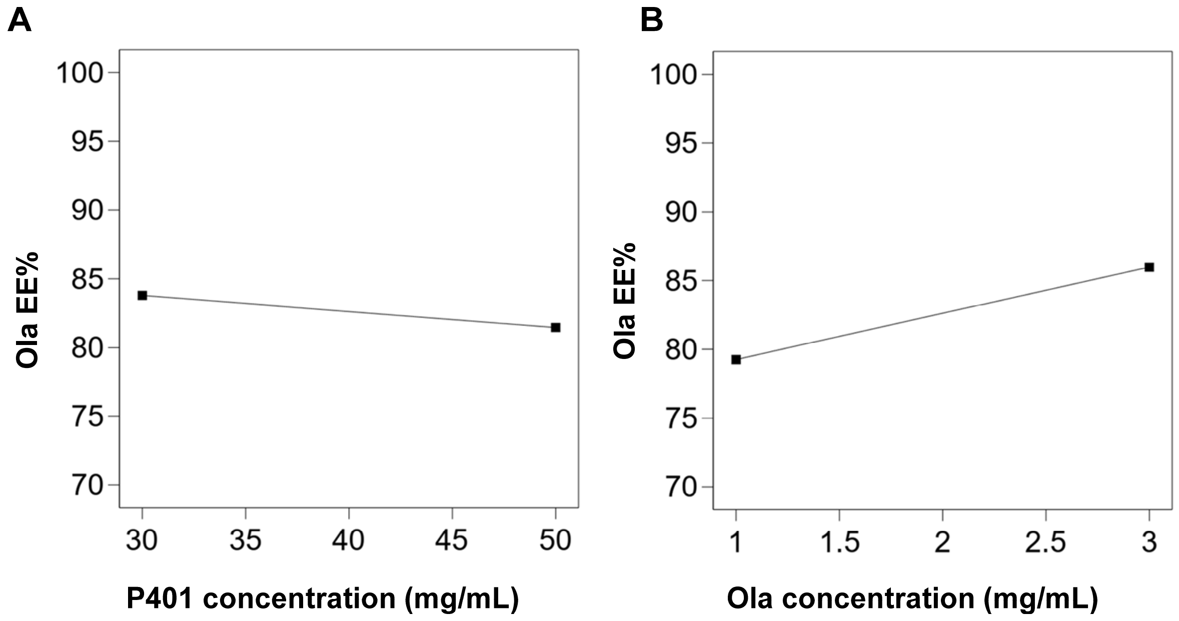

Figure S2. The effect of different significant variables on olanzapine loaded polymersomes EE% (Y2). (A) Increasing P401 concentration decreases EE%. (B) Ola concentration has a positive effect on EE%.

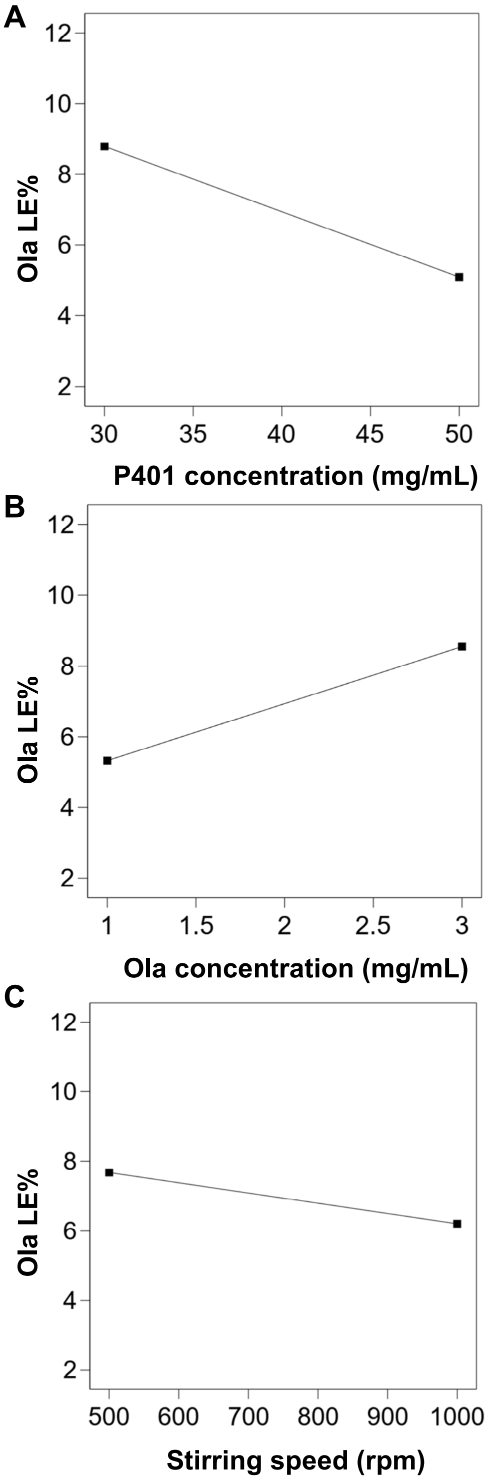

Figure S3. The effect of different significant variables on olanzapine loaded polymersomes LE% (Y3). (A) increasing P401 concentration decreased the LE%. (B) Ola concentration had a positive influence on LE%. (C) $Poly_{Ola}$ LE% is inversely proportional to stirring speed.

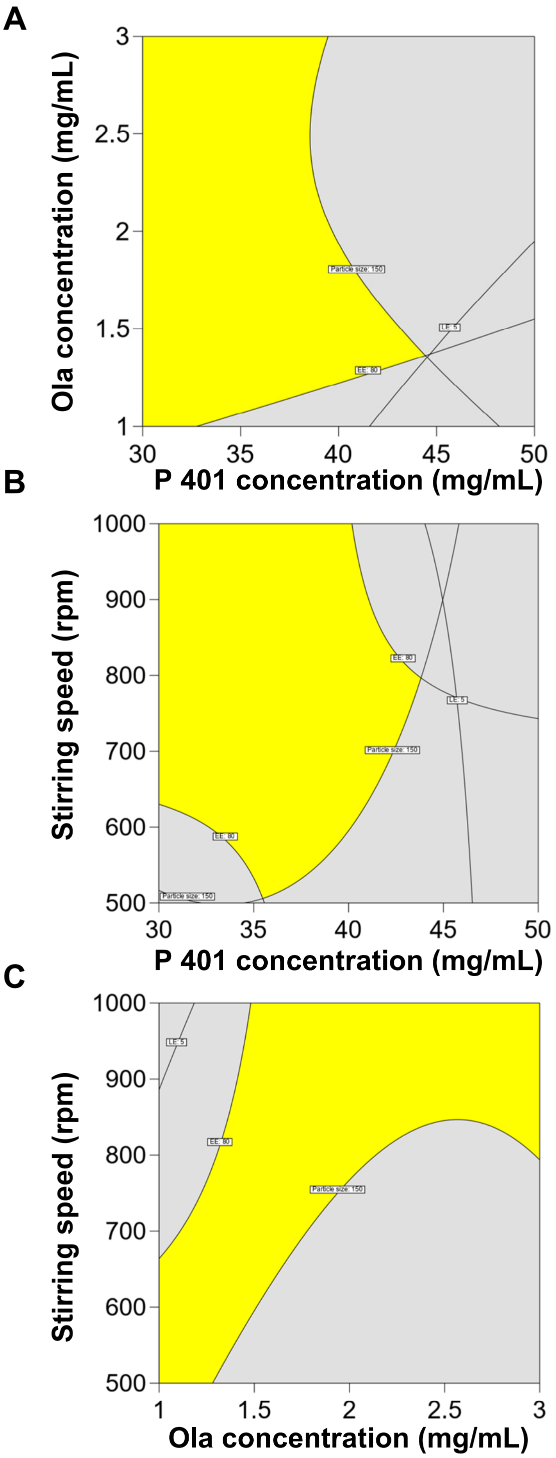

Figure S4. Overlay plots depicting the design space region for the optimized Poly$_{Ola}$. The design space was plotted by overlapping variables contour plots to obtain required responses. The yellow area represents the values of variables when optimized to fulfill optimization criteria; minimum particle size, maximum EE % and LE%.

Table S8. The experimental and predicted physicochemical characterization of the optimized polymersomes ($Poly_{Ola}$) [a]

Parameter	Experimental	Predicted	% Predicted error
Particle size (nm) [b, e]	78.3± 4.5	80.23	2.41
EE (%) [c, e]	91.36± 3.55	95.15	4.14
LE (%) [d, e]	9.11± 1.59	9.58	5.15

[a] $Poly_{Ola}$ is composed of poloxamer 401 (30 mg/mL) and olanzapine (3 mg/mL) stirred at 850 rpm.
[b] measured by dynamic light scattering technique after dilution in deionized water (1: 100 v/v).
[c] calculated directly as percentage of olanzapine added, determined by HPLC.
[d] calculated as percentage of entrapped Ola weight to total Poly weight
[e] expressed as mean ±SD.
